# Differential plasma protein expression after ingestion of essential amino acid-based dietary supplement verses whey protein in low physical functioning older adults

**DOI:** 10.1007/s11357-023-00725-5

**Published:** 2023-02-01

**Authors:** Gohar Azhar, Ambika Verma, Xiaomin Zhang, Amanda Pangle, Pankaj Patyal, Wei Zhang, Yingni Che, Karen Coker, Robert R. Wolfe, Jeanne Y. Wei

**Affiliations:** 1 Department of Geriatrics, Donald W. Reynolds Institute On Aging, University of Arkansas for Medical Sciences, 4301 West Markham, Little Rock, AR 72205 USA; 2 Department of Mathematics and Statistics, University of Arkansas at Little Rock, Little Rock, AR 72204 USA

**Keywords:** Essential amino acids, Whey protein, Nutrition, Aging, Protein expression

## Abstract

In a recent randomized, double-blind, placebo-controlled trial, we were able to demonstrate the superiority of a dietary supplement composed of essential amino acids (EAAs) over whey protein, in older adults with low physical function. In this paper, we describe the comparative plasma protein expression in the same subject groups of EAAs vs whey. The plasma proteomics data was generated using SOMA scan assay. A total of twenty proteins were found to be differentially expressed in both groups with a 1.5-fold change. Notably, five proteins showed a significantly higher fold change expression in the EAA group which included adenylate kinase isoenzyme 1, casein kinase II 2-alpha, Nascent polypeptide-associated complex subunit alpha, peroxiredoxin-1, and peroxiredoxin-6. These five proteins might have played a significant role in providing energy for the improved cardiac and muscle strength of older adults with LPF. On the other hand, fifteen proteins showed slightly lower fold change expression in the EAA group. Some of these 15 proteins regulate metabolism and were found to be associated with inflammation or other comorbidities. Gene Ontology (GO) enrichment analysis showed the association of these proteins with several biological processes. Furthermore, protein–protein interaction network analysis also showed distinct networks between upregulated and downregulated proteins. In conclusion, the important biological roles of the upregulated proteins plus better physical function of participants in the EAAs vs whey group demonstrated that EAAs have the potential to improve muscle strength and physical function in older adults. This study was registered with ClinicalTrials.gov: NCT03424265 “Nutritional interventions in heart failure.”

## Introduction

The expression of proteins in blood changes as a function of age, health conditions, environmental exposures, nutrition, and dietary supplements. Protein is key dietary ingredient, especially for older adults who undergo age-related loss of muscle mass which contributes to low physical functioning (LPF). We and others have previously reported that older adults tend to underestimate their protein requirements and eat substantially less protein [1, 2]. It can be very challenging to reverse the rate of decline in physical functioning with aging [3, 4]. To be able to live independently at an older age, it is very important to maintain optimal skeletal muscle function and strength. Pharmacological approaches for ameliorating LPF in the elderly have not been successful due to multiple comorbidities associated with age and side effects of drugs [5]. In some cases, exercise training has improved the functional performances of older peoples with LPF [6, 7], but not in all [8, 9].

Nutritional therapies such as whey protein and essential amino acids (EAAs) have both produced health benefits [10–12]. One advantage of EAAs over whey is that they are primary “active” components of dietary protein responsible for the stimulation of muscle protein synthesis [10]. In fact, the response of muscle protein synthesis after ingestion of a composition of free EAAs has been reported to be more than twice the response to ingestion of a comparable dosage (g/g) of whey protein isolate [11, 13]. Various previous studies have found that supplementation of the diet with EAAs increases lean body mass, muscle strength, and improved physical function by effectively stimulating muscle protein synthesis in the elderly [14–17]. Moreover, another study that used EAA supplementation in older adults showed promising results in the moderate reduction of plasma triglycerides, which are an independent risk factor for ischemic heart disease [18].

There is very limited data evaluating the differential expression of proteins in blood following consumption of EAAs vs whey in older individuals. An improved understanding of the differential expression of proteins secondary to EAAs or whey could help to inform the design of appropriate nutritional therapies for LPF older adults. Evaluation of plasma proteins can provide important diagnostic, prognostic, and potentially therapeutic information. However, it is very challenging to quantify the proteins because of required dynamic range to profile the human proteome which contains of an approximately 20,000 proteins [19]. In a recent study, we described that the 12 weeks of dietary supplementation with a specially formulated composition based on EAAs improved physical performance in LPF elderly as compared to whey protein and could be considered an effective therapy in individuals with low physical functional capacity [7]. In this companion paper, we used SOMAscan [20], to study differential plasma protein expression in the same cohort of older adults with LPF and showed that five proteins had a significantly higher expression in the EAA group which included adenylate kinase isoenzyme 1(AK1), casein kinase II 2-alpha, Nascent polypeptide-associated complex subunit alpha (NACA), peroxiredoxin-1, and peroxiredoxin-6. These five proteins, plus some inflammatory proteins that were differentially reduced, might have contributed to the better physical performance of the LPF subjects on EAAs.

## Methods

### Subjects

This was a randomized double-blind, placebo-controlled study, conducted at the University of Arkansas for Medical Sciences. The study was approved by IRB# 206,313. Sixty subjects were randomized to either EAAs or whey over a 12-week period. Inclusion criteria were age ≥ 65, both genders, and inclusive of all races and ethnicities as described [7]. The primary criterion for participation was difficulty with performing physical activity for reasons other than orthopedic limitations (i.e., low physical function, LPF). All our subjects had some level of previously diagnosed degree of cardiac disease and were clinically, New York Heart Association (NYHA) I-III, with either heart failure with preserved ejection fraction (HFpEF) or systolic heart failure. We used the subjects with NYHA level I, II and III symptoms and most subjects were either level I or level II. The characteristics of the subjects are summarized in Table 1.

**Table 1:** Characteristics of the subjects of EAA and whey protein groups

Categories	Specific factor	Whey protein *N* = 20	EAAs *N* = 16
NYHA class	NYHA class 1	3	2
	NYHA class 2	16	12
	NYHA class 3	2	2
	NYHA class 4	0	0
Echocardiography	EF ≥ 60%	19	14
	EF ≤ 40–55%	1	1
Other comorbidities	HTN	19	15
	HLP	14	8
	Atrial fibrillation	4	3
	CAD	3	4
	DM	2	1

The following were included in the exclusion criteria: hemoglobin < 10 g/dL; eGFR < 30; hemoglobin A1c ≥ 10; allergy to milk or soy products; inability to perform strength and/or functional assessments; moderate to severe heart valve disease; myocardial infarction in the past 6 months; infiltrative, restrictive, or hypertrophic cardiomyopathy; unstable angina; dementia, as determined by a SLUMS score of < 20; active inflammatory bowel disease; having received chemotherapy or radiation therapy within the past 12 months; currently undergoing tube feeding; currently receiving palliative care for end-of-life circumstance; and those who were not willing to refrain from using non-study protein/amino acid supplements during their participation in this study. In addition, potential subjects with any disease that specifically impaired functional capacity were excluded, such as moderate hypothyroidism, Parkinson’s disease, major anxiety/depression, myasthenia gravis, and neurological diseases that cause gait impairment (e.g., amyotrophic lateral sclerosis, stroke). A total of 60 subjects participated in the trial but plasma for SOMAscan analysis was obtained from 20 subjects on whey and 16 subjects on EAAs. SOMAscan is a highly multiplexed, sensitive platform that uses modified DNA aptamers as high affinity protein capture reagents to simultaneously quantify more than 1300 human proteins in plasma [20].

### *Experimentaldesign*

#### Overview

Two groups of subjects were randomly assigned to consume daily for 12 weeks one of two different nutritional supplements, a proprietary EAA composition, or whey protein isolate. Blood samples were collected from subjects at baseline and at the end of the study.

#### Nutritional supplements

Participants receiving nutritional supplementation were instructed to consume a daily dose of 15 g of the proprietary EAA-based composition (US Patent 9,597,367 B2) or 15 g of a whey protein isolate composition daily for 12 weeks. The EAA composition contained histidine, isoleucine, leucine, lysine, methionine, phenylalanine, threonine, valine, tryptophan, citrulline, and carnitine (12 g total per dose) and 3 g of non-caloric flavoring.

The whey protein isolate composition contained approximately 13.5 g whey protein isolate (90% protein) and 1.5 g flavoring. Products were supplied by The Amino Company, LLC, Lewes, Delaware. Subjects and investigators were blinded about which product they were taking. All product containers were coded and after all study visits were completed, the blind was broken, and all data were entered into a database.

Participants were asked to maintain their normal diet and given a food diary for daily recording upon enrollment. Dietary data was analyzed using special computer software (Esha Research Inc, 4747 Skyline RD, Ste. 10, Salem, OR 97,306) and no significant differences were found between the macronutrient intake of the EAAs and whey protein groups. Dietary protein intake was an average of 0.85 ± 0.06 g protein/kg/day in the EAA group, and 0.87 ± 0.050 g in the whey group [7].

#### Study visits (participants in randomized double-blind trial)

##### Visits 1 and 2

Informed consent was obtained, the SLUMS cognitive test was administered and scored, a history and physical performed, and a blood sample obtained. On the 2nd visit, a fasting blood sample (~ 30 mL) was drawn to determine baseline values and subjects were randomized in a double-blind method. A permuted block randomization procedure, stratified by gender, was used to assign subjects to EAAs or whey group. Subjects were provided with forms and training for recording their dietary intake over a 3-day period each week. Study staff dispensed approximately 10 days’ worth of study product and a consumption diary for subjects to complete after ingesting each dose.

##### Visits 3–13 (study weeks 1–11 of intervention)

Review of any adverse effects, diet review, compliance with supplements, and more study product provided.

##### Visit 7 (study week 6)

Participants reported after an overnight fast and a blood sample (~ 30 ml) was drawn. A urine sample was collected for a dipstick test for proteinuria.

##### Visit 13 (study week 12)

Procedures performed in visit 7 were repeated.

The primary functional measure was the distance walked in 6 min or 6-min walk (6 MW) distance at baseline and final visit in all participants in both whey and EAA groups. In addition, body composition was determined at baseline and final visit of participants in both groups by dual-energy X-ray absorptiometry. Blood plasma samples were processed and analyzed commercially, and statistical data analysis performed to examine the differential protein expression in the plasma of subjects taking EAAs vs whey. Blood samples were collected in lavender potassium EDTA tubes, and then spun down to separate out the plasma. Plasma was then aliquoted into flip-top PCR tubes and shipped frozen on dry ice, Washington University (WashU, St. Louis, MO) for SOMALogic analysis.

### Statistical analysis

Summary statistics included transforming and visualizing the proteomics data came from SOMAscan analysis, Washington University (WashU, St. Louis, MO). The hypothesis was that there was a significant difference between protein expression with respect to treatments. Firstly, fold change ratios of protein expression before and after treatment were calculated and transformed on a log2 scale to make all asymmetric values to be symmetric around zero and distribution of ratios were observed in terms of positive, negative or no change. Then, data normalization was done to reduce the variation arising from treatment vs control. Furthermore, to calculate the differences in protein expression between EAAs and whey groups, Welch two sample t-test was performed. Volcano plots and heatmaps were generated by using a 1.5-fold change in expression between EAAs and whey groups. Dendrogram was prepared to see the clustered analysis of identified proteins in both groups. Statistical analyses were performed using R 4.1.2.

The 6 MW (ft) change from baseline at 12 weeks was tested for the null hypotheses of no change per group and no group difference in change. For the per-group tests, the one-sample t-test was used for the Whey group and the Wilcoxon signed rank test for the EAA group. The group comparison was tested using the Mann–Whitney *U* test. The alternative hypotheses, a positive change per group and a greater change for the EAA group compared to the Whey group, were one-sided. The significance level was 0.05. Statistical analysis was performed using SAS 9.4.

### Transforming and visualizing proteomics data

The proteomics data was transformed to a log2 scale, to observe ratios that were positive or negative after 12 weeks of treatment. A value larger than 1 indicates that protein was expressed higher after treatment. Data was normalized for run-to-run variation [21] and missing values were removed. We performed 1305 t-tests using Welch Two sample t-test. Proteins were extracted by using log2 fold change greater than 0.585 or less than − 0.585 (which means 1.5-fold change expression), and a log10 of p-values greater than 1.3.

## Results

**Fig. 1:**
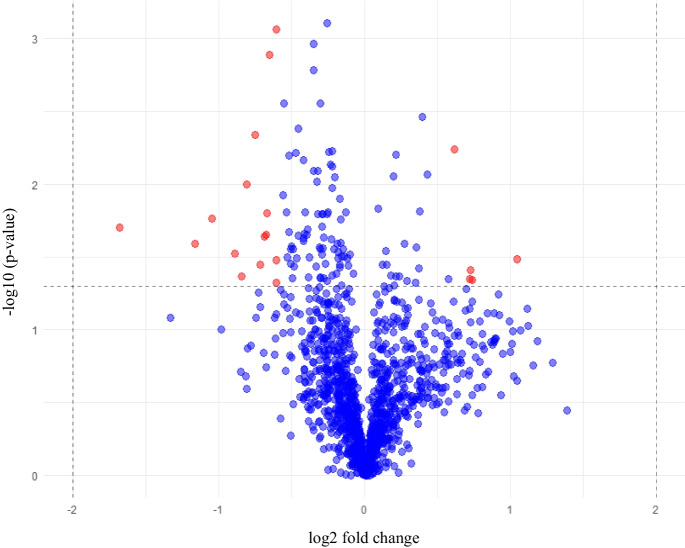
Volcano plot showed the differential protein expression in the blood plasma samples obtained from older adults after 12 weeks of EAAs vs whey protein consumption using a 1.5-fold change expression. Red dots on the right-hand side indicate upregulation and on the left-hand side indicate downregulation and black dots indicate no significant change in protein expression levels based on an absolute log2 fold change greater than 0.585 or less than − 0.585, indicates 1.5-fold change expression with − log10 *p*-value greater than 1.3

**Fig. 2:**
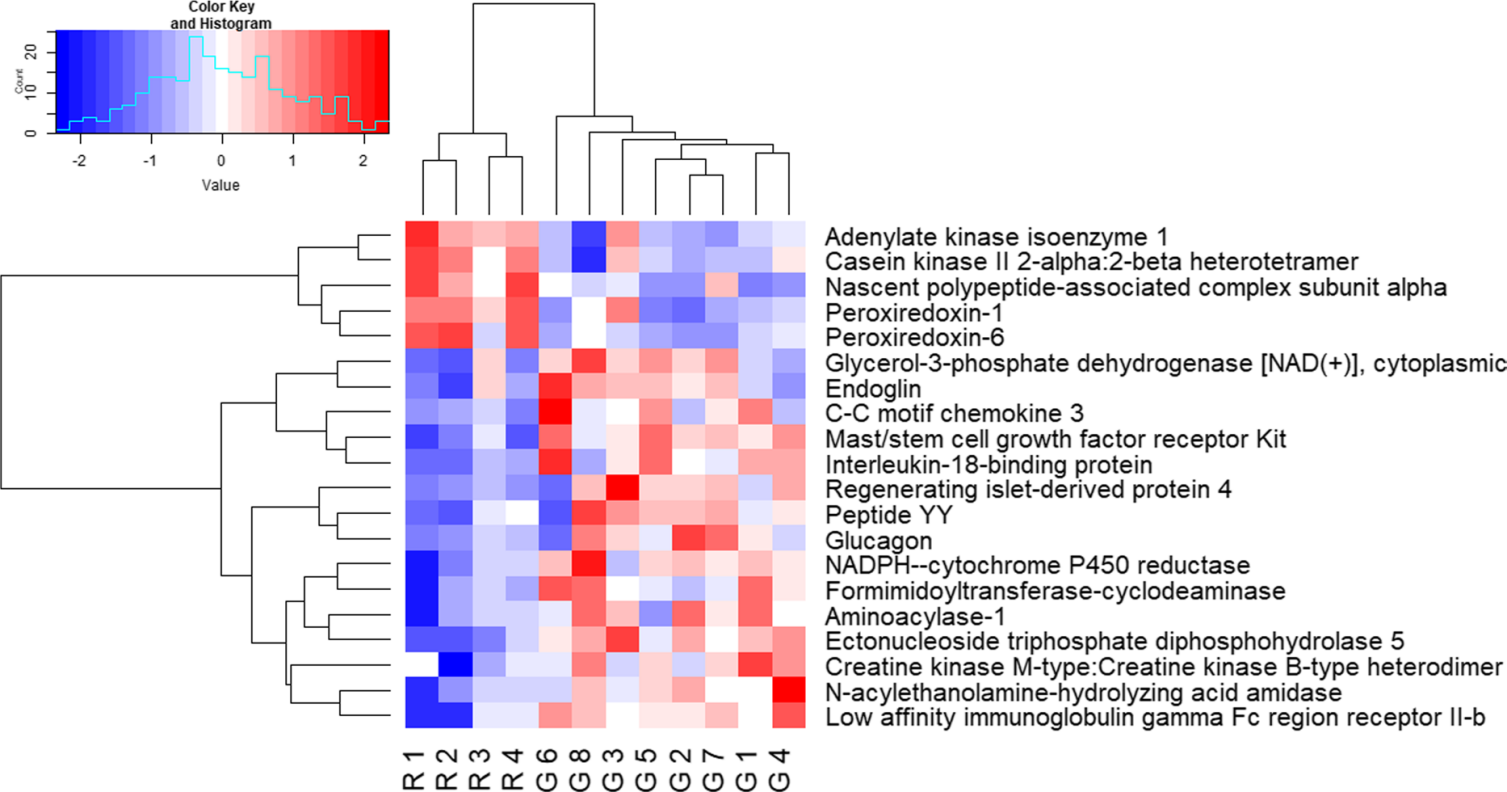
Heat map plot for the expression of the differentially expressed proteins in EAAs and whey group in which red color represents the high value and blue color as low value

**Fig. 3:**
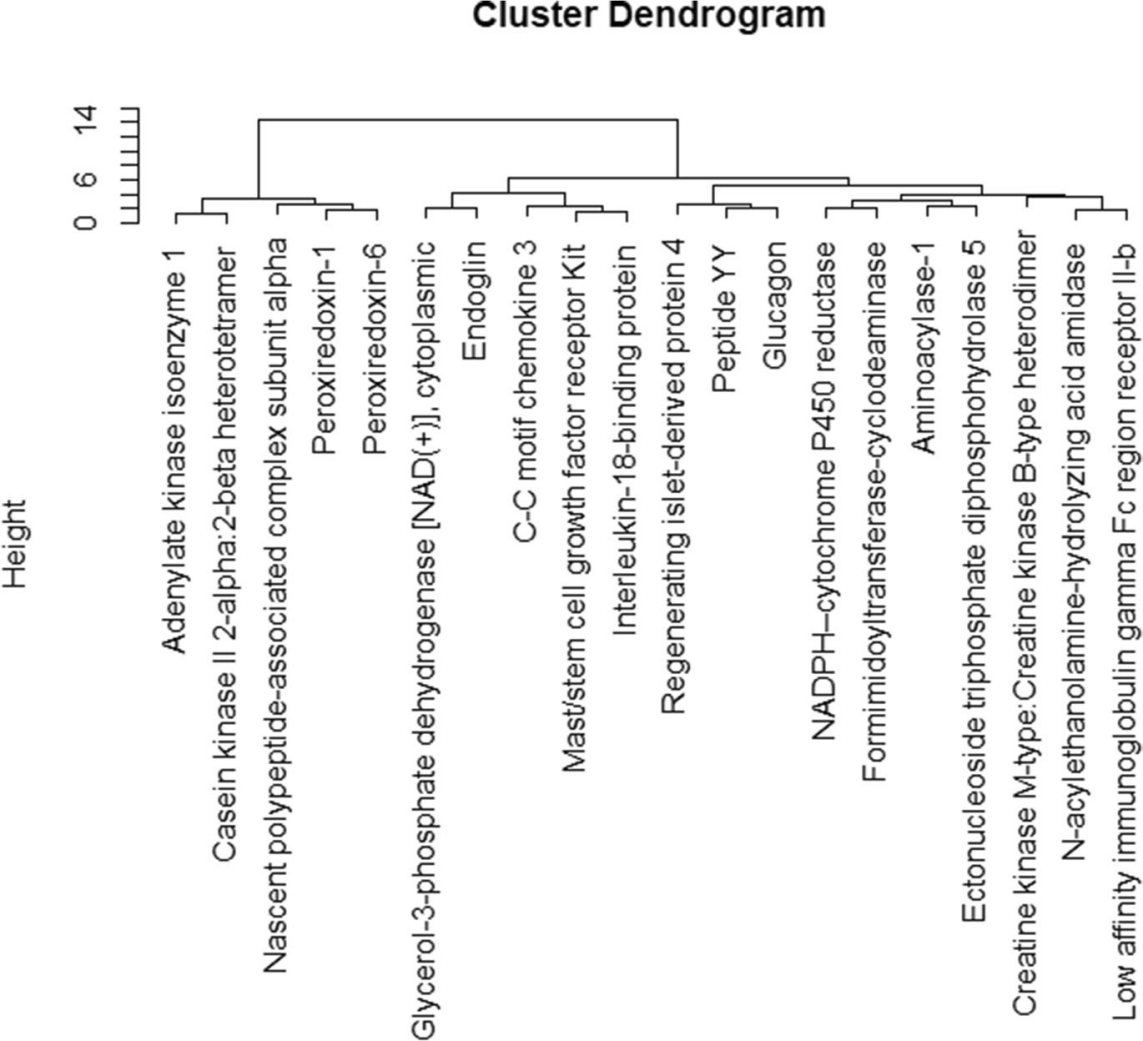
Dendrogram showing clustered analysis of significantly expressed proteins in EAAs and whey group

A total of twenty proteins were differentially expressed between subjects taking EAAs vs whey after 12 weeks (Table 2) and we observed that five proteins had a higher fold change in EAAs vs whey groups (Table 2, Fig. 1). Differential protein expression is shown in the heatmap (Fig. 2), and protein clusters in the dendrogram (Fig. 3). Body composition/anthropometric changes, before and after consuming EAAs and whey protein are presented in Table 3.

**Table 2:** Most differentially expressed proteins using a 1.5-fold change (EAAs vs whey group), log2 fold change > 0.585 (upregulated), <  − 0.585 (downregulated), and − log10 *p* > 1.3 (significant)

Differentially Expressed Proteins in EAAs vs whey group		Log2 fold change (EAA vs whey)	− Log10 of *p*-value	Fold change (EAA vs whey)	Effect size
A: upregulated proteins (EAAs vs whey group)					
1.	Adenylate kinase isoenzyme 1	1.05	1.49	2.071	2.08
2.	Casein kinase II 2-alpha:2-beta heterotetramer	0.729	1.41	1.657	2.23
3.	Nascent polypeptide-associated complex subunit alpha	0.740	1.34	1.670	2.30
4.	Peroxiredoxin-1	0.607	2.24	1.524	2.17
5.	Peroxiredoxin-6	0.721	1.35	1.648	2.75
B: downregulated proteins (EAAs vs whey group)					
6.	Glycerol-3-phosphate dehydrogenase [NAD( +)], cytoplasmic	− 0.888	1.52	0.540	1.37
7.	Endoglin	− 0.608	1.33	0.656	1.23
8.	C–C motif chemokine 3	− 0.718	1.45	0.608	1.10
9.	Mast/stem cell growth factor receptor Kit	− 0.654	2.89	0.636	2.81
10.	Interleukin-18-binding protein	− 0.753	2.34	0.593	1.68
11.	Regenerating islet-derived protein 4	− 0.687	1.64	0.621	1.22
12.	Peptide YY	− 0.607	1.48	0.657	1.25
13.	Glucagon	− 1.16	1.59	0.447	1.19
14.	NADPH–cytochrome P450 reductase	− 1.05	1.76	0.483	1.32
15.	Formimidoyltransferase-cyclodeaminase	− 1.68	1.70	0.312	1.26
16.	Aminoacylase-1	− 0.808	2.00	0.571	1.54
17.	Ectonucleoside triphosphate diphosphohydrolase 5	− 0.606	3.07	0.657	2.48
18.	Creatine kinase M-type:Creatine kinase B-type heterodimer	− 0.847	1.37	0.556	1.24
19.	N-acylethanolamine-hydrolyzing acid amidase	− 0.677	1.65	0.625	1.27
20.	Low affinity immunoglobulin gamma Fc region receptor II-b	− 0.667	1.80	0.630	2.07

**Table 3:** Body composition/anthropometric changes before and after consuming EAAs and whey protein

	EAAs (*n* = 16)			Whey (*n* = 20)		
	Baseline	Final	*p*-values	Baseline	Final	*p*-values
	Mean ± SEM	Mean ± SEM		Mean ± SEM	Mean ± SEM	
Body weight (kg)	81.85 ± 3.85	80.75 ± 3.93	0.4285	82.09 ± 3.3	81.28 ± 3.18	0.4341
BMI (mg/m^2^)	29.88 ± 1.13	29.55 ± 1.16	0.4147	30.38 ± 1.34	30.07 ± 1.25	0.4471
Lean body mass (kg)	45.12 ± 2.61	45.21 ± 2.77	0.9242	44.91 ± 1.78	45.42 ± 1.89	0.3888
Fat mass (kg)	32.57 ± 2.04	31.88 ± 2.02	0.3443	32.73 ± 2.19	32.02 ± 2.89	0.2785

*BMI*, body mass index; *EAAs*, essential amino acids

### Gene Ontology (GO) enrichment analysis of differentially expressed proteins

**Fig. 4:**
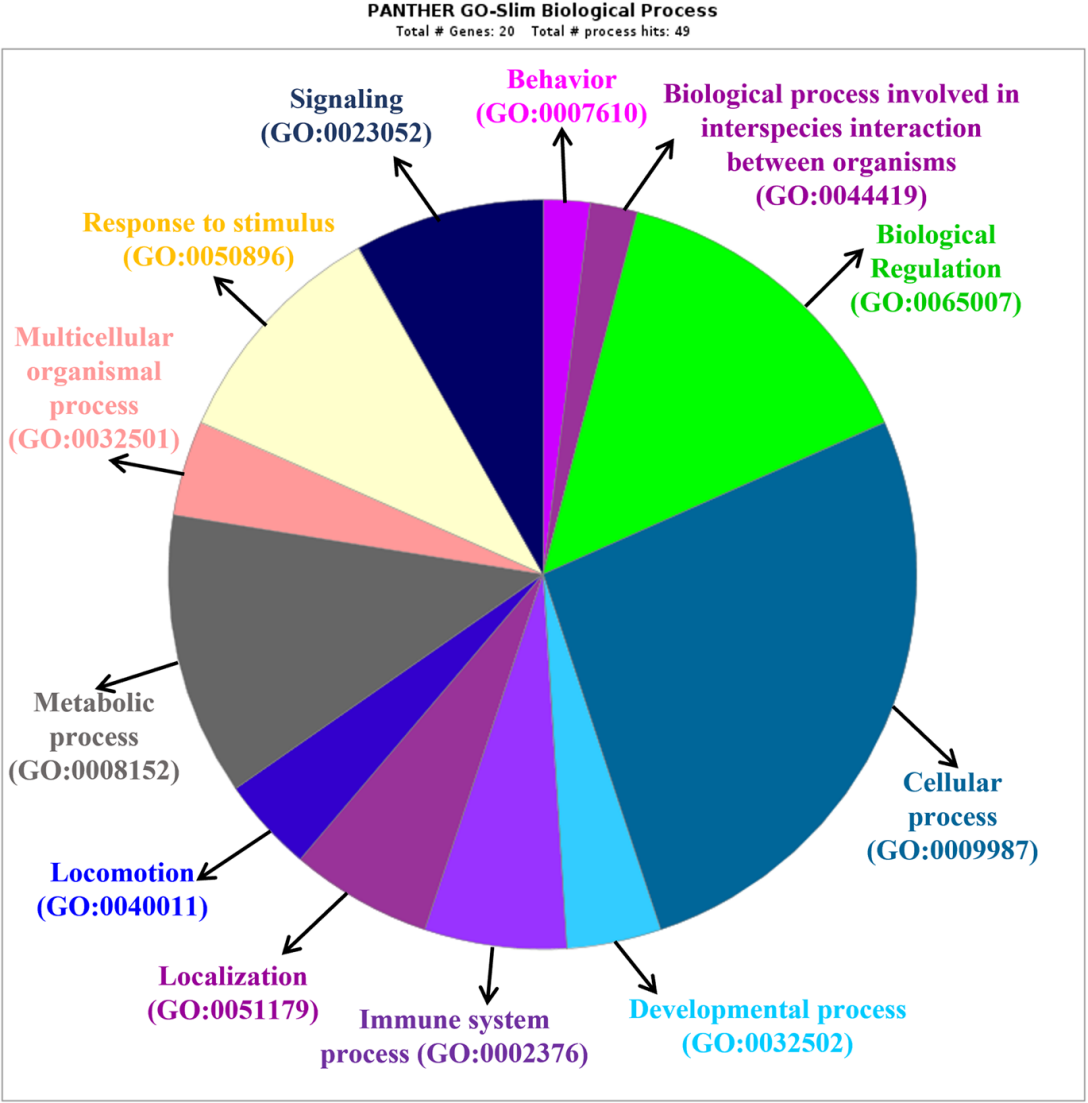
Pie Chart showing the functional classification of differentially expressed proteins in EAAs vs whey group using Gene Ontology (GO) enrichment analysis through PANTHER-classification system

All the differentially expressed proteins were mapped to their enriched GO terms based on the functional annotation from the PANTHER database (Fig. 4). The majority of protein class are associated with cellular processes (13 proteins), biological regulations (7 proteins) and metabolic processes (6 proteins). In the upregulated five proteins, three out of five are associated with biological regulations, viz., Casein kinase II 2-alpha:2-beta heterotetramer, Peroxiredoxin-1, and Peroxiredoxin-6, and two out of five are associated with metabolic process, viz., Adenylate kinase isoenzyme 1 and Peroxiredoxin-1.

Of the 15 downregulated proteins, 8 proteins are found to be associated with cellular processes, viz., Glycerol-3-phosphate dehydrogenase [NAD( +)] cytoplasmic, Endoglin, C–C motif chemokine 3, Mast/stem cell growth factor receptor Kit, Peptide YY, Ectonucleoside triphosphate diphosphohydrolase 5, Creatine kinase M-type: Creatine kinase B-type heterodimer, and Low affinity immunoglobulin gamma Fc region receptor II-b, and four out of 15 are associated with biological regulation, viz., C–C motif chemokine 3, Mast/stem cell growth factor receptor Kit, Peptide YY, and Low affinity immunoglobulin gamma Fc region receptor II-b, and again four proteins are found to be associated with metabolic process, viz., Glycerol-3-phosphate dehydrogenase [NAD( +)], cytoplasmic, Mast/stem cell growth factor receptor Kit, Ectonucleoside triphosphate diphosphohydrolase 5 and Creatine kinase M-type: Creatine kinase B-type heterodimer.

### PPI network analysis of the differentially expressed proteins

**Fig. 5:**
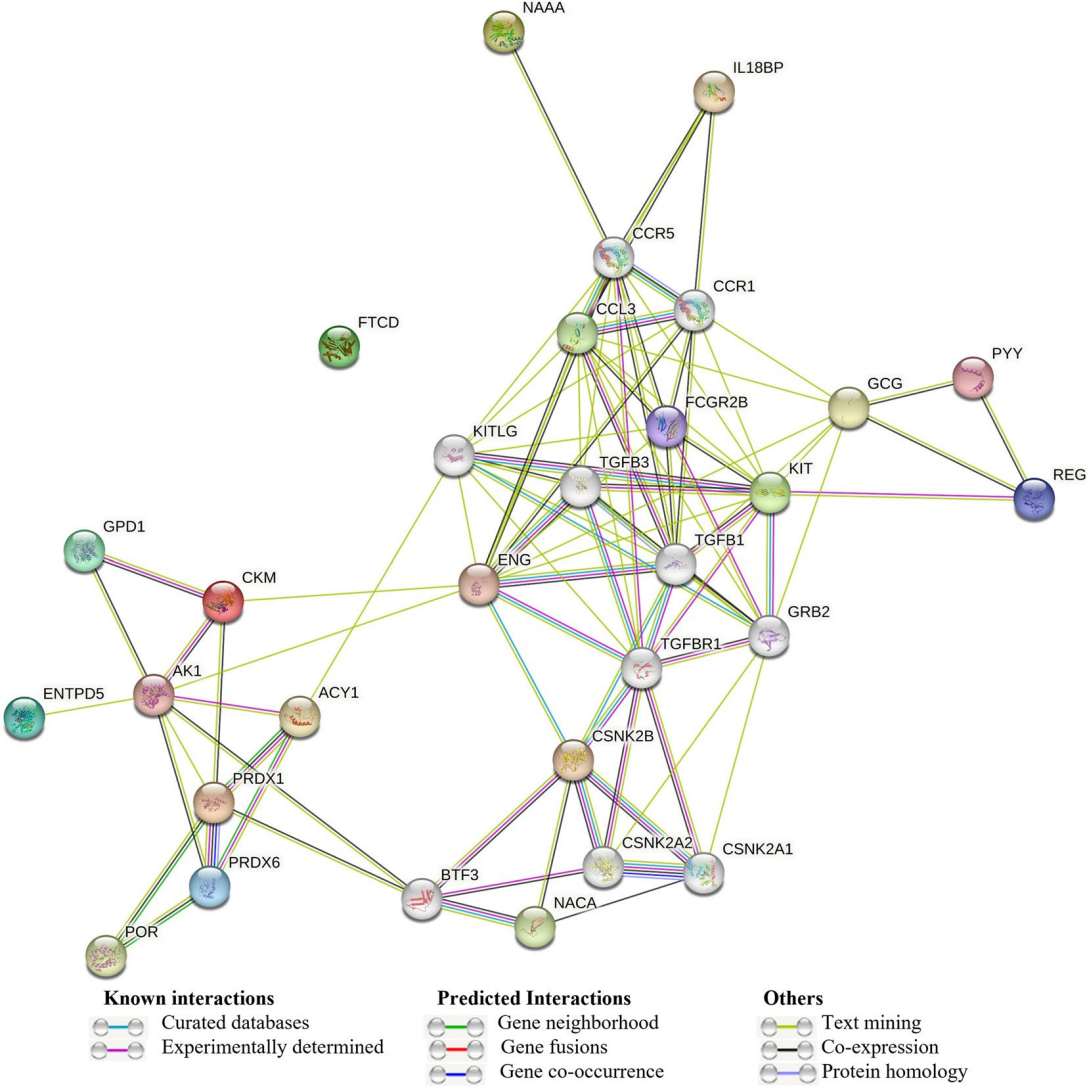
Protein–protein interactions using STRING database among all the 20 differentially expressed proteins extracted from 1.5-fold change expression among EAAs vs whey groups

The protein–protein interaction network analysis was constructed using the STRING database for the upregulated and downregulated categories of proteins (Fig. 5). A total of 5 differentially expressed proteins in the upregulated group and 15 differentially expressed proteins in the downregulated group were found to show interactions in the matched PPI networks. In the upregulated ones, two distinct interactions were found and among which, two proteins, viz., Peroxiredoxin-1 and Peroxiredoxin-6, presented the highest degree of connectivity as shown in Fig. 5. In the downregulated ones, three distinct interactive networks were found and four proteins, viz., Endoglin, C–C motif chemokine 3, Low affinity immunoglobulin gamma Fc region receptor II-b, and Mast/stem cell growth factor receptor Kit, presented the highest degree of connectivity as shown in Fig. 5.

### Functional analysis of change in 6MWT distance

**Fig. 6:**
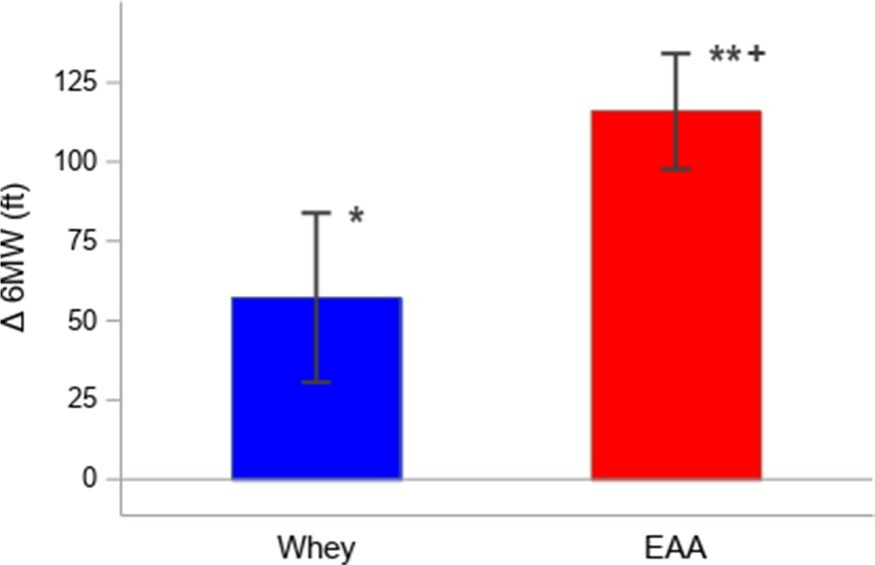
6 MW (ft) Change from baseline: mean ± SE. *p < 0.05 by the one-sample *t* test for the whey group’s 6 MW (ft) change from baseline 12 weeks. **p < 0.01 by the signed rank test for the EAA group’s 6 MW (ft) change from baseline 12 weeks. + *p* < 0.01 by the Mann–Whitney *U* test for the group comparison of change from baseline at 12 weeks

The mean distance (SE) traveled by the EAA group during baseline was 1261.5 (76.5) ft vs 1422.9 (56.6) ft by the whey group. As shown in Fig. 6, both groups increased the mean distance traveled at the final visit by 115.9 (18.2) ft, *p* < 0.01, in the EAA group and 57.3 (26.6) ft, *p* < 0.05, in the whey group. Furthermore, while the whey group traveled a longer distance at both the baseline and final visit than the EAA group, the magnitude of change in the EAA group from baseline to the final visit was significantly different from the whey group (*p* < 0.01).

## Discussion

Our previous research has found the health benefits of nutritional diet supplement with EAAs based composition vs whey protein in older adults. This is the first study which shows comparative protein expression between EAAs vs whey proteins supplementation in LPF elders and relates it to physical functional improvement.

The major finding of this study was that daily supplementation of the diet with the EAAs compared to whey showed a significantly higher expression level in five key proteins that might have contributed to the better muscle strength and improvement in 6 min walking distance in LPF older adults. Adenylate kinase isoenzyme 1 (AK1), showed the greatest fold change difference in EAAs vs whey. AK1 is an important regulator of cellular energy expenditure, and it has been found to be highly expressed in well-differentiated tissues with high energy demand, such as brain, skeletal muscle and heart, and found to be critical in the maintenance of cellular and muscle performance [22–26]. More recently, a modest elevation of AK1 activity has been shown to improve functional recovery following ex-vivo model of ischemia–reperfusion in murine hearts [27]. Other investigators have reported a fourfold decrease in expression of AK1 in a mouse model of Duchene’s muscle atrophy [28]. Interestingly, AK1 protein expression has also been shown to be elevated in the skeletal muscle of old vs young adult rats but this could represent a compensatory increase [29]. The elevation of AK1 in our study might have contributed to increased cytosolic availability of cyclic AMP improving muscle energetics.

Higher expression of Casein kinase II 2-alpha:2-beta heterotetramer (CK2) with EAAs might also have been beneficial since CK2 has been found to regulate cell survival in various tissues including muscles cells, and also controls muscle cells during physiological aging [30–34]. Another important protein elevated with EAAs was Nascent polypeptide-associated complex subunit alpha (NACA). NACA has been shown to be crucial for cardiomyocyte growth as well as skeletal muscle growth and regeneration. Knock-down of skeletal NACA (skNAC) in zebrafish embryos resulted in skeletal muscle defects [35–38].

Peroxiredoxin-1and 6 (Prdx1 and Prdx6) were also higher in EAAs vs whey. Prdx-1 plays an important role in cell protection against oxidative stress by detoxifying peroxides and as sensor of hydrogen peroxide-mediated signaling events [39]. It has been reported that the overexpression of Prdx1 in cardiomyocytes of mice prevented transverse aortic constriction (TAC)-induced cardiac hypertrophy and heart failure [40, 41]. Prdx-6 has been reported to play an important role in heart recovery following ischemia–reperfusion injury and can protect against phospholipid peroxidation-mediated membrane damage [42–44]. These two proteins, viz., Prdx-1 and Prdx-6, also presented the highest degree of connectivity as per the STRING network analysis of protein–protein interactions (Fig. 5).

These five proteins of EAA group were found to be clustered together in dendrogram and shared a significantly close cellular processes and biological regulations as per protein analysis through evolutionary relationships (PANTHER) classification system (Fig. 4). These biological functions of higher fold expressed five proteins in EAAs supported our previous study that these five proteins were helpful to provide additional energy for cardiac and muscle strength in EAAs supplemented older adults as compared to whey group in which these proteins showed slightly lower fold change in expression. These results also support our previous findings that EAAs supplements have the potential to improve muscle strength and physical function in LPF older people as compared to whey groups [7].

Fifteen proteins were found to have slightly lower fold change expression in EAAs vs whey group. This decrease in expression in these proteins was relatively mild, between 0.3 and 0.65-fold change. Some of these 15 proteins are important as they regulates metabolic processes of body like Glycerol-3-phosphate dehydrogenase [NAD( +)], cytoplasmic, possesses glycerol-3-phosphate dehydrogenase activity and involved in important cellular glycolytic processes. Glycerol-3-phosphate 1-like proteins are found to be highly expressed in heart tissue, with lower levels in the skeletal muscle, kidney, lung, and other organs [45]. It has been reported that mitochondrial Glycerol-3-phosphate dehydrogenase, regulates myoblast differentiation and contributes to the process of skeletal muscle regeneration, and deficiency of its expression was identified in the skeletal muscles of patients and animal models of obesity and diabetes [46]. Another important protein, Glucagon has a crucial role in the maintenance of heart function, as it is considered to be a cardio stimulant agent that increases heart rate and contractility, and it has been used as a therapeutic for heart failure treatments [47, 48]. But its increased levels have been found to suppress skeletal muscle protein synthesis, which attenuates the ability of skeletal muscle to synthesize proteins that may evolve into sarcopenia [49], and have also been considered deleterious in type 2 diabetes [47]. This mild decrease of Glucagon levels in our study might have contributed to improved muscle function and walking ability in LPF older adults on EAA.

NADPH-cytochrome P450 reductase, is another essential enzyme which is known to be well expressed in the heart, where it may participate in the metabolism of therapeutic agents and environmental toxicants [50]. Cardiac P450 enzymes has been found to play a critical role in cardiac ischemia–reperfusion injury, through increases in the production of free radicals [51]. It has also been reported that deletion of NADPH-cytochrome P450 reductase gene in cardiomyocytes does not protect mice against doxorubicin-mediated acute cardiac toxicity [52].

Another crucial protein is Creatine kinase M-type: Creatine kinase B-type heterodimer, (CK-MB), which plays a central role in energy transduction in tissues with large, fluctuating energy demands, such as skeletal muscle, heart, and brain. In muscle cells, this extra energy buffer plays a pivotal role in maintaining ATP homeostasis [53]. Its expression levels have been reported to be elevated in myocardial infarction (MI), myocarditis, pericarditis, muscular dystrophy, cardiac defibrillation, cardiac surgery, extensive rhabdomyolysis, strenuous exercise (marathon runners), mixed connective tissue disease, cardiomyopathy, and hypothermia [54]. Mild lower expression of CK-MB in our study, might have been beneficial for LPF for cardiovascular health of LPF older adults.

Some proteins which showed lower fold expression in EAAs vs whey groups, support our study for improved health with EAAs in LPF elder people because these proteins have been found to show higher expressions in severe heart diseases, like C–C motif chemokine 3 (CCL3), a monokine with inflammatory and chemokinetic properties, which has been found to be strongly related to myocardial ischemia as it is elevated in patients with acute myocardial infarction and unstable angina pectoris [55]. Interleukin-18-binding protein, (IL-18) is a member of the IL-1 family of cytokines and increasing numbers of clinical studies indicate a role for IL-18 in heart diseases. Previous studies also reported an increased expression of IL-18 in circulating T cells of patients with ischemic and dilated cardiomyopathy [56], and upregulation of IL-18 mRNA after myocardial infarction in mice [57]. Endoglin, has also been reported to be highly expressed in human hearts with severe left ventricular systolic dysfunction and with major adverse cardiovascular events like congestive heart failure, acute myocardial infarction, stroke, and sudden cardiac death [58]. Peptide YY (PYY), has been found to be associated with parameters of cardiovascular risk as well as cardiovascular events and mortality in patients presenting with acute myocardial infarction [59].

There are several other proteins which play critical roles in controlling several human diseases and showed slightly lower fold expression in EAAs vs whey groups, which includes Formimidoyltransferase-cyclodeaminase (FTCD), a folate-dependent enzyme which has been found to be a key factor for downregulating mTORC1 activity under the circumstances of fasting mediating starvation response and protecting the human health [60]. Ectonucleoside triphosphate diphosphohydrolase 5 (ENTPD5) is a soluble enzyme that hydrolyzes purine nucleoside diphosphates and involved in protein glycosylation pathway, which is part of protein modification. ENTPD5 has been reported to be an essential player for skeletal mineralization and regulates phosphate homeostasis in zebrafish [61]. It has also been found that ENTPD5 deficient mice develop progressive Hepatopathy, Hepatocellular tumors and spermatogenic arrest [62]. Aminoacylase-1 (ACY1) controls the cytosolic breakdown of acetylated amino acids produced during protein degradation. It is known that its deficiency can cause neurological problems and individuals with this condition typically have delayed development of mental and motor skills (psychomotor delay) [63]. Regenerating islet-derived protein 4 (REG4), is a secretory protein which play an important role in cell differentiation and proliferation. Its expression has been found to be upregulated in inflammatory bowel diseases and in many gastrointestinal malignancies which ultimately end up with colorectal and gastric carcinomas [64–66]. Recently, it has been reported that Reg4 protein along with Reg3 protein directs accumulation of functionally distinct macrophages subsets after myocardial infarction [67]. Mast/stem cell growth factor receptor Kit helps in the regulation of cell survival and proliferation, stem cell maintenance, and mast cell development etc. and mutations in this gene are associated with various gastrointestinal stromal tumors, mast cell disease, acute myelogenous leukemia, and piebaldism. It has been reported that significant proportion of acral, mucosal and vulvar melanomas have KIT mutations [68, 69].

Some proteins which appear to be important in severe inflammatory diseases are Low affinity immunoglobulin gamma Fc region receptor II-b (FcγRIIB), which is the only inhibitory Fc receptor that controls many aspects of immune and inflammatory responses, and it is one of the genes thought to influence susceptibility to several autoimmune diseases in humans [70]. The elevated levels of FcγRIIB expression have been reported in the vascular tissue of mouse hypertensive models, and blockade of FcγRIIB function significantly reduced Ang II (angiotensin II)–induced vascular remodeling and hypertension in mice [71]. N-acylethanolamine-hydrolyzing acid amidase (NAAA) is one of the enzymes that take part in the hydrolysis of *N*-acylethanolamines (NAEs) e.g., N-palmitoylethanolamine, which are bioactive lipids, involved in many physiological processes including pain, inflammation, anxiety, cognition, and food intake and therefore, regulate their endogenous levels and effects [72]. Recent studies showed that NAAA inhibition appears to be beneficial in severe physiological conditions related to inflammation and pain [72, 73]. All these fifteen proteins showed slightly lower fold change in expression in EAAs vs whey protein group, which might not have had functional consequences. In the downregulated proteins in EAAs vs whey group, four proteins, viz., Endoglin, C–C motif chemokine 3, Low affinity immunoglobulin gamma Fc region receptor II-b and Mast/stem cell growth factor receptor Kit, presented the highest degree of connectivity as per STRING network analysis of protein–protein interactions (Fig. 5).

**Fig. 7:**
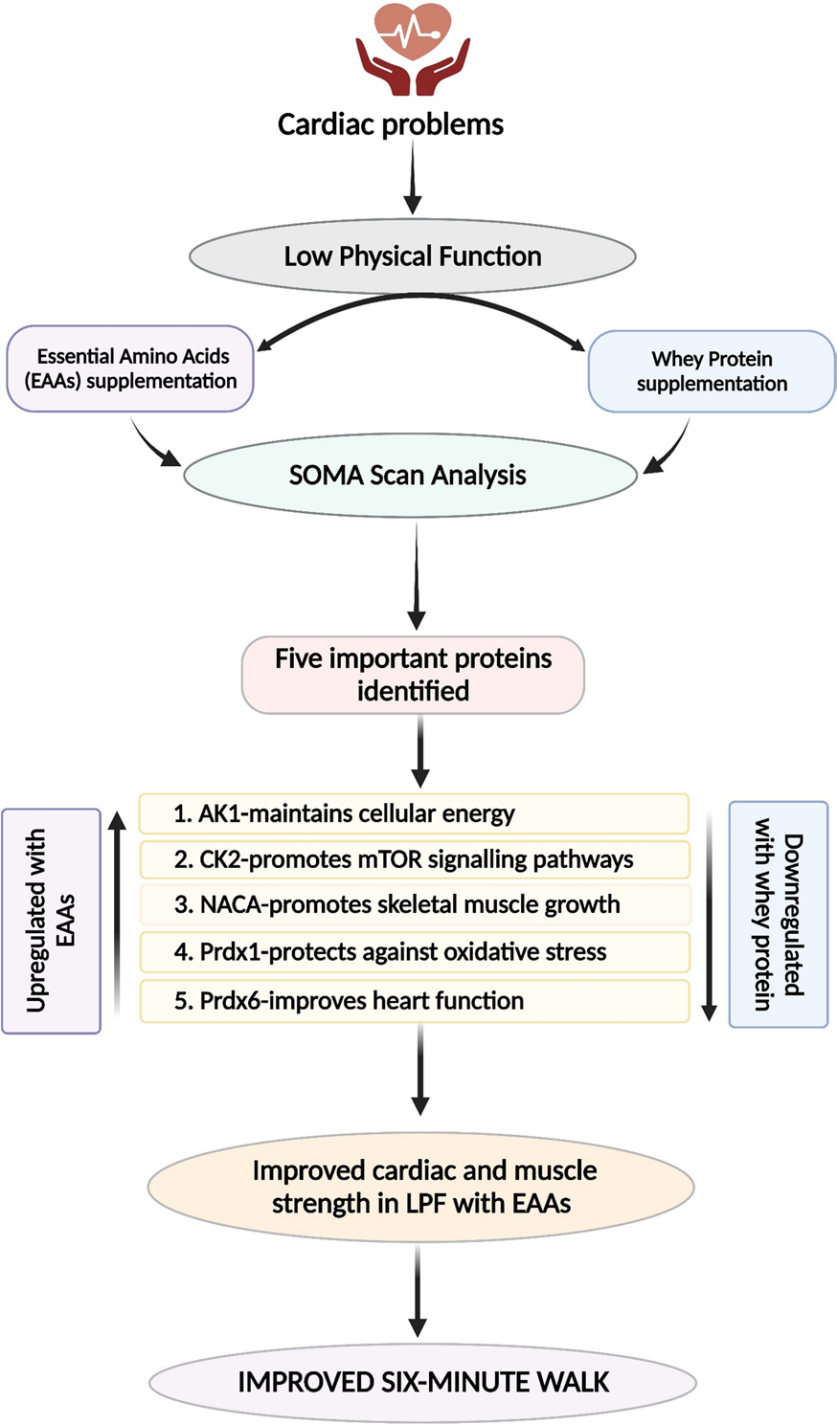
Schematic illustrating the process by which EAA supplementation improved physical functioning in older adults

The principal finding of this study was that there were five proteins which showed a higher fold expression after consumption of EAAs by LPF older individuals vs whey proteins and each one of these proteins has a significant role in providing energy which might have improved muscle strength and performance in 6 MW distance (Fig. 7). As we discussed here, some of the proteins expressed slightly lower in EAAs vs whey group were found to be important for regulating metabolic processes of body, but rest were found to show higher expression in various disease states. These results are in line with previous studies in which nutritional supplementation of the diet in low physical functioning older individuals with specially formulated composition based on EAAs provide enhanced physical function as compared to supplementation with the equal amount of whey protein. This conclusion also supports the results of similar studies evaluating the effect of supplementation of the diet with EAAs on low physical functioning older subjects.

## Data Availability

The data generated in this study are available from the corresponding authors upon reasonable request.

## References

[1] Picetti D, Foster S, Pangle A, Schrader A, Wei JY, Azhar G (2017). Knowledge and consumption of protein in older adults: Opportunity for improvement. Int J Dev Res.

[2] Cheng YL, Sung SH, Cheng HM, Hsu PF, Guo CY, Yu WC, Chen CH (2017). Prognostic nutritional index and the risk of mortality in patients with acute heart failure. J Am Heart Assoc.

[3] Kitzman DW, Nicklas B, Kraus WE, Lyles MF, Eggebeen J, Morgan TM, Haykowsky M (2014). Skeletal muscle abnormalities and exercise intolerance in older patients with heart failure and preserved ejection fraction. Am J Physiol-Heart Circ Physiol.

[4] Azhar G, Wei JY (2015). The demographics of aging and its impact on the cardiovascular health. Curr Cardiovasc Risk Rep.

[5] Braunstein JB, Anderson GF, Gerstenblith G (2003). Noncardiac comorbidity increases preventable hospitalizations and mortality among Medicare beneficiaries with chronic heart failure. J Am Coll Cardiol.

[6] Gary RA, Sueta CA, Dougherty M (2004). Home-based exercise improves functional performance and quality of life in women with diastolic heart failure. Heart Lung.

[7] Azhar G, Wei JY, Schutzler SE, Coker K, Gibson RV, Kirby MF, Ferrando AA, Wolfe RR (2021). Daily Consumption of a Specially Formulated Essential Amino Acid-Based Dietary Supplement Improves Physical Performance in Older Adults with Low Physical Functioning. J Gerontol A Biol Sci Med Sci.

[8] Baum JI, Kim IY, Wolfe RR (2016). Protein consumption and the elderly: What is the optimal level of intake?. Nutrients.

[9] Maldonado-Martín S, Brubaker PH, Eggebeen J, Stewart KP, Kitzman DW (2017). Association between 6-minute walk test distance and objective variables of functional capacity after exercise training in elderly heart failure patients with preserved ejection fraction: a randomized exercise trial. Arch Phys Med Rehabil.

[10] Volpi E, Kobayashi H, Sheffield-Moore M, Mittendorfer B, Wolfe RR (2003). Essential amino acids are primarily responsible for the amino acid stimulation of muscle protein anabolism in healthy elderly adults. Am J Clin Nutr.

[11] Baum JI, Wolfe RR (2015). The Link between Dietary Protein Intake, Skeletal Muscle Function and Health in Older Adults. Healthcare.

[12] Kim IY, Park S, Smeets ET, Schutzler S, Azhar G, Wei JY, Ferrando AA, Wolfe RR (2019). Consumption of a specially formulated mixture of essential amino acids promotes gain in whole-body protein to a greater extent than a complete meal replacement in older women with heart failure. Nutrients.

[13] Paddon-Jones D, Sheffield-Moore M, Katsanos CS, Zhang XJ, Wolfe RR (2006). Differential stimulation of muscle protein synthesis in elderly humans following isocaloric ingestion of amino acids or whey protein. Exp Gerontol.

[14] Volpi E, Ferrando AA, Yeckel CW, Tipton KD, Wolfe RR (1998). Exogenous amino acids stimulate net muscle protein synthesis in the elderly. J Clin Investig.

[15] Paddon-Jones D, Sheffield-Moore M, Zhang XJ, Volpi E, Wolf SE, Aarsland A, Ferrando AA, Wolfe RR (2004). Amino acid ingestion improves muscle protein synthesis in the young and elderly. Am J Physiol Endocrinol Metab.

[16] Dillon EL, Sheffield-Moore M, Paddon-Jones D (2009). Amino acid supplementation increases lean body mass, basal muscle protein synthesis, and insulin-like growth factor-I expression in older women. J Clin Endocrinol Metab.

[17] Børsheim E, Bui QU, Tissier S (2009). Amino acid supplementation decreases plasma and liver triacylglycerols in elderly. Nutrition.

[18] Marquis BJ, Hurren NM, Carvalho E, Kim IY, Schutzler S, Azhar G, Wolfe RR, Børsheim E (2017). Skeletal Muscle Acute and Chronic Metabolic Response to Essential Amino Acid Supplementation in Hypertriglyceridemic Older Adults. Curr Dev Nutr.

[19] Gold L, Ayers D, Bertino J, Bock C, Bock A (2010). Aptamer-Based Multiplexed Proteomic Technology for Biomarker Discovery. PLoS ONE.

[20] Kim CH, Tworoger SS, Stampfer MJ, Dillon ST, Gu X, Sawyer SJ, Chan AT, Libermann TA, Eliassen AH (2018). Stability and reproducibility of proteomic profiles measured with an aptamer-based platform. Sci Rep.

[21] Bolstad BM, Irizarry RA, Astrand M, Speed TP (2003). A comparison of normalization methods for high density oligonucleotide array data based on variance and bias. Bioinformatics (Oxford, England).

[22] Janssen E, Dzeja PP, Oerlemans F, Simonetti AW, Heerschap A, De Haan A, Rush PS, Terjung RR, Wieringa B, Terzic A (2000). Adenylate kinase 1 gene deletion disrupts muscle energetic economy despite metabolic rearrangement. EMBO J.

[23] Hancock CR, Janssen E, Terjung RL (2005). Skeletal muscle contractile performance and ADP accumulation in adenylate kinase deficient mice. Am J Physiol Cell Physiol.

[24] Dzeja PP, Bast P, Pucar D, Wieringa B, Terzic A (2007). Defective Metabolic Signaling in Adenylate Kinase AK1 Gene Knock-out Hearts Compromises Post-Ischemic Coronary Reflow. J Biol Chem.

[25] Dzeja PP, Chung S, Faustino RS, Behfar A, Terzic A (2011). Developmental Enhancement of Adenylate Kinase-AMPK Metabolic Signaling Axis Supports Stem Cell Cardiac Differentiation. PLoS ONE.

[26] Yegutkin GG, Wieringa B, Robson SC, Jalkanen S (2012). Metabolism of circulating ADP in the bloodstream is mediated via integrated actions of soluble adenylate kinase-1 and NTPDase1/CD39 activities. FASEB J.

[27] Sevasti Z (2021). Subtle Role for Adenylate Kinase 1 in Maintaining Normal Basal Contractile Function and Metabolism in the Murine Heart. Front Physiol.

[28] Yue Ge (2003). Proteomic analysis of mdx skeletal muscle: Great reduction of adenylate kinase 1 expression and enzymatic activity. Proteomics.

[29] O’Connell, Kathleen, et al. Proteomic and Biochemical Profiling of Aged Skeletal Muscle. Sarcopenia–Age-Related Muscle Wasting and Weakness. Springer, Dordrecht. 2011; 259–287.

[30] Meggio F, Pinna LA (2003). One-thousand-and-one substrates of protein kinase CK2?. FASEB J.

[31] Salvi M, Sarno S, Cesaro L, Nakamura H, Pinna LA (2009). Extraordinary pleiotropy of protein kinase CK2 revealed by weblogo phosphoproteome analysis. Biochim Biophys Acta.

[32] Götz C, Montenarh M (2017). Protein kinase CK2 in development and differentiation. Biomed Rep.

[33] Nuñez de Villavicencio-Diaz T, Rabalski AJ, Litchfield DW (2017). Protein Kinase CK2: intricate relationships within regulatory cellular networks. Pharmaceuticals.

[34] Salizzato V, Zanin S, Borgo C, Lidron E, Salvi M, Rizzuto R, Pallafacchina G, Donella-Deana A (2019). Protein kinase CK2 subunits exert specific and coordinated functions in skeletal muscle differentiation and fusogenic activity. FASEB J.

[35] Wiedmann B, Sakai H, Davis TA, Wiedmann M (1994). A protein complex required for signal-sequence-specific sorting and translocation. Nature.

[36] Sims RJ, Weihe EK, Zhu L, O’Malley S, Harriss JV, Gottlieb PD (2002). m-Bop, a repressor protein essential for cardiogenesis, interacts with skNAC, a heart- and muscle-specific transcription factor. J Biol Chem.

[37] Li H, Randall WR, Du SJ (2009). SkNAC (skeletal Naca), a muscle-specific isoform of Naca (nascent polypeptide-associated complex alpha), is required for myofibril organization in zebrafish embryo. FASEB J.

[38] Park CY, Pierce SA, von Drehle M, Ivey KN, Morgan JA, Blau HM, Srivastava D (2010). skNAC, a Smyd1-interacting transcription factor, is involved in cardiac development and skeletal muscle growth and regeneration. Proc Natl Acad Sci U S A.

[39] Wadley AJ, Aldred S, Coles SJ (2016). An unexplored role for Peroxiredoxin in exercise-induced redox signaling?. Redox Biol.

[40] Tang C, Yin G, Huang C, Wang H, Gao J, Luo J, Zhang Z, Wang J, Hong J, Chai X (2020). Peroxiredoxin-1 ameliorates pressure overload-induced cardiac hypertrophy and fibrosis. Biomed Pharmacother.

[41] Jiang L, Gong Y, Hu Y, You Y, Wang J, Zhang Z, Wei Z, Tang C. Peroxiredoxin-1 overexpression attenuates doxorubicin-induced cardiotoxicity by inhibiting oxidative stress and cardiomyocyte apoptosis. Oxid Med. Cell Longev*.* 2020; 2405135. 10.1155/2020/2405135 PMC7411498 32802259

[42] Manevich Y, Sweitzer T, Pak JH, Feinstein SI, Muzykantov V, Fisher AB (2002). 1-cys peroxiredoxin overexpression protects cells against phospholipid peroxidation-mediated membrane damage. Proc Natl Acad Sci USA.

[43] Nagy N, Malik G, Fisher AB, Das DK (2006). Targeted disruption of peroxiredoxin 6 gene renders the heart vulnerable to ischemia-reperfusion injury. Am J Physiol Heart Circ Physiol.

[44] Li DX, Chen W, Jiang YL, Ni JQ, Lu L (2020). Antioxidant protein peroxiredoxin 6 suppresses the vascular inflammation, oxidative stress, and endothelial dysfunction in angiotensin ii-induced endotheliocyte. Gen Physiol Biophys.

[45] London B, Michalec M, Mehdi H, Zhu X, Kerchner L, Sanyal S, Viswanathan PC, Pfahnl AE, Shang LL, Madhusudanan M, Baty CJ, Lagana S, Aleong R, Gutmann R, Ackerman MJ, McNamara DM, Weiss R, Dudley SC (2007). Jr Mutation in glycerol-3-phosphate dehydrogenase 1 like gene (GPD1-L) decreases cardiac Na+ current and causes inherited arrhythmias. Circulation.

[46] Liu X, Qu H, Zheng Y, Liao Q, Zhang L, Liao X, Xiong X, Wang Y, Zhang R, Wang H, Tong Q, Liu Z, Dong H, Yang G, Zhu Z, Xu J, Zheng H (2018). Mitochondrial glycerol 3-phosphate dehydrogenase promotes skeletal muscle regeneration. EMBO Mol Med.

[47] Ceriello A, Genovese S, Mannucci E, Gronda E (2016). Glucagon and heart in type 2 diabetes: new perspectives. Cardiovasc Diabetol.

[48] Petersen KM, Bogevig S, Holst JJ, Knop FK, Christensen MB (2018). Hemody- namic efects of glucagon: a literature review. J Clin Endocrinol Metab.

[49] Adeva-Andany MM, Fernández-Fernández C, López-Pereiro Y, Castro-Calvo I, Carneiro-Freire N (2021). The effects of glucagon and the target of rapamycin (TOR) on skeletal muscle protein synthesis and age-dependent sarcopenia in humans. Clinical nutrition ESPEN.

[50] Wang JF, Yang Y, Sullivan MF, Min J, Cai J, Zeldin DC, Xiao YF, Morgan JP (2002). Induction of cardiac cytochrome p450 in cocaine-treated mice. Exp Biol Med (Maywood).

[51] Granville DJ, Tashakkor B, Takeuchi C, Gustafsson AB, Huang C, Sayen MR, Wentworth P, Yeager M, Gottlieb RA (2004). Reduction of ischemia and reperfusion-induced myocardial damage by cytochrome P450 inhibitors. Proc Natl Acad Sci USA.

[52] Fang C, Gu J, Xie F, Behr M, Yang W, Abel ED, Ding X (2008). Deletion of the NADPH-cytochrome P450 reductase gene in cardiomyocytes does not protect mice against doxorubicin-mediated acute cardiac toxicity. Drug Metab Dispos.

[53] Hettling H, van Beek JH (2011). Analyzing the functional properties of the creatine kinase system with multiscale ‘sloppy’ modeling. PLoS Comput Biol.

[54] Fred F Ferri. C-Laboratory Tests and Interpretation of Results. Ferri’s Clinical Advisor. 2022;1951–1967

[55] Saskia CA, de Jager, Adriaan O Kraaijeveld, Robert W Grauss, Wilco de Jager, Su-San Liem, Bas L van der Hoeven, Berent J Prakken, Hein Putter, Theo JC, van Berkel, Douwe E. Atsma, Martin J Schalij, J Wouter Jukema, Erik AL Biessen. CCL3 (MIP-1α) levels are elevated during acute coronary syndromes and show strong prognostic power for future ischemic events. J Mol Cell Cardiol. 2008; 45 (3):446-452 10.1016/j.yjmcc.2008.06.003 18619972

[56] Yndestad A, Holm AM, Muller F, Simonsen S, Froland SS, Gullestad L, Aukrust P (2003). Enhanced expression of inflammatory cytokines and activation markers in T-cells from patients with chronic heart failure. Cardiovasc Res.

[57] Woldbaek PR, Tonnessen T, Henriksen UL, Florholmen G, Lunde PK, Lyberg T, Christensen G (2003). Increased cardiac IL-18 mRNA, pro-IL-18 and plasma IL-18 after myocardial infarction in the mouse; a potential role in cardiac dysfunction. Cardiovasc Res.

[58] Shyu K-G (2017). The Role of Endoglin in Myocardial Fibrosis. Acta Cardiol Sin.

[59] Haj-Yehia E, Mertens RW, Kahles F, Rückbeil MV, Rau M, Moellmann J, Biener M, Almalla M, Schroeder J, Giannitsis E, Katus HA, Marx N, Lehrke M (2020). Peptide YY (PYY) Is Associated with Cardiovascular Risk in Patients with Acute Myocardial Infarction. J Clin Med.

[60] Zhang W, Wu C, Ni R, Yang Q, Luo L, He J (2021). Formimidoyltransferase cyclodeaminase prevents the starvation-induced liver hepatomegaly and dysfunction through downregulating mTORC1. PLoS Genet.

[61] Huitema LF, Apschner A, Logister I, Spoorendonk KM, Bussmann J, Hammond CL, Schulte-Merker S (2012). Entpd5 is essential for skeletal mineralization and regulates phosphate homeostasis in zebrafish. Proc Natl Acad Sci USA.

[62] Read R, Hansen G, Kramer J, Finch R, Li L, Vogel P (2009). Ectonucleoside triphosphate diphosphohydrolase type 5 (Entpd5)-deficient mice develop progressive hepatopathy, hepatocellular tumors, and spermatogenic arrest. Vet Pathol.

[63] Engelke UF, Sass JO, Van Coster RN, Gerlo E, Olbrich H, Krywawych S, Calvin J, Hart C, Omran H, Wevers RA (2008). NMR spectroscopy of aminoacylase 1 deficiency, a novel inborn error of metabolism. NMR Biomed.

[64] Violette S, Festor E, Pandrea-Vasile I, Mitchell V, Adida C, Dussaulx E (2003). Reg IV, a new member of the regenerating gene family, is overexpressed in colorectal carcinomas. Int J Cancer.

[65] Oue N, Mitani Y, Aung PP, Sakakura C, Takeshima Y, Kaneko M (2005). Expression and localization of Reg IV in human neoplastic and non-neoplastic tissues: reg IV expression is associated with intestinal and neuroendocrine differentiation in gastric adenocarcinoma. J Pathol.

[66] Saukkonen K, Hagström J, Mustonen H, Lehtinen L, Carpen O, Andersson LC, Seppänen H, Haglund C (2018). Prognostic and diagnostic value of REG4 serum and tissue expression in pancreatic ductal adenocarcinoma. Tumour Biol.

[67] Lörchner H, Hou Y, Adrian-Segarra JM, Kulhei J, Detzer J, Günther S, Gajawada P, Warnecke H, Niessen HW, Pöling J, Braun T (2018). Reg proteins direct accumulation of functionally distinct macrophage subsets after myocardial infarction. Cardiovasc Res.

[68] Yun J, Lee J, Jang J, Lee EJ, Jang KT, Kim JH, Kim KM (2011). KIT amplification and gene mutations in acral/mucosal melanoma in Korea. APMIS Acta Pathologica, Microbiologica, Et Immunologica Scandinavica.

[69] Omholt K, Grafström E, Kanter-Lewensohn L, Hansson J, Ragnarsson-Olding BK (2011). KIT pathway alterations in mucosal melanomas of the vulva and other sites. Clin Cancer Res.

[70] Smith KG, Clatworthy MR (2010). FcgammaRIIB in autoimmunity and infection: evolutionary and therapeutic implications. Nat Rev Immunol.

[71] Song X, Zou X, Ge W, Hou C, Cao Z, Zhao H, Zhang T, Jin L, Fu Y, Kong W, Yan C, Cai J, Wang J (2021). Blocking FcγRIIB in Smooth Muscle Cells Reduces Hypertension. Circ Res.

[72] Bottemanne P, Muccioli GG, Alhouayek M (2018). N-acylethanolamine hydrolyzing acid amidase inhibition: tools and potential therapeutic opportunities. Drug Discovery Today.

[73] Gorelik A, Gebai A, Illes K, Piomelli D, Nagar B (2018). Molecular mechanism of activation of the immunoregulatory amidase NAAA. Proc Natl Acad Sci USA.

