# Correction to: Differential plasma protein expression after ingestion of essential amino acid-based dietary supplement versus whey protein in low physical functioning older adults

**DOI:** 10.1007/s11357-023-00751-3

**Published:** 2023-02-10

**Authors:** Gohar Azhar, Ambika Verma, Xiaomin Zhang, Amanda Pangle, Pankaj Patyal, Wei Zhang, Yingni Che, Karen Coker, Robert R. Wolfe, Jeanne Y. Wei

**Affiliations:** 1 Department of Geriatrics, Donald W. Reynolds Institute On Aging, University of Arkansas for Medical Sciences, 4301 West Markham, Little Rock, AR 72205 USA; 2 Department of Mathematics and Statistics, University of Arkansas at Little Rock, Little Rock, AR 72204 USA

**Correction to: GeroScience**

10.1007/s11357-023-00725-5

Unfortunately, the authors for this published article noticed an error in the article title.

The word verses in the article title should be corrected to versus. The corrected article title is shown above.

